# Only a boost away from re-entry

**DOI:** 10.1007/s12471-017-0991-2

**Published:** 2017-04-24

**Authors:** E. Ströker, C. de Asmundis, G. B. Chierchia, P. Brugada

**Affiliations:** Heart Rhythm Management Centre, UZ Brussel-VUB, Brussels, Belgium

## Answer

**Fig. 1:**
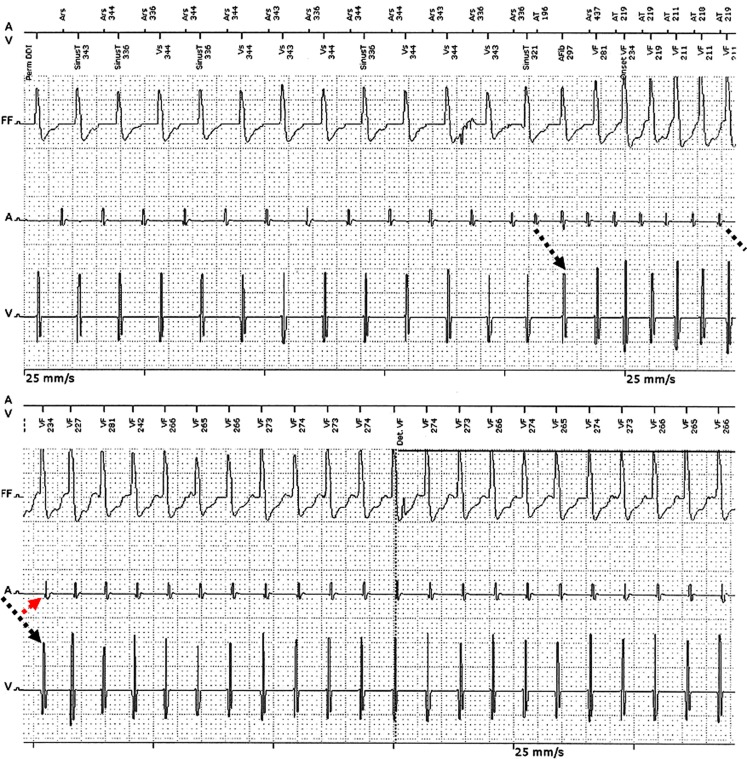
The episode showed a sequence of sinus tachycardia (during exercise), followed by a few beats of PACs with a ‘jump’ into the slow pathway (*black dashed line*) after the first beat, further PR interval prolongation with crossing over until PR of 320 ms after the 8^th^ beat. That last beat could be conducted retrogradely into the fast atrioventricular nodal pathway (echo beat, *red dashed line*), initiating AVNRT (*PACs* Premature atrial contractions, *AVNRT* atrioventricular nodal reentrant tachycardia)

Looking closely, the dual atrioventricular nodal (AVN) physiology is evident with a ‘jump’ into the slow pathway (SP) and a crossing over phenomenon, before retrograde conduction over the fast pathway (FP) initiating typical AVN reentrant tachycardia (AVNRT) (Fig. 1). A new electrophysiological study (EPS) was performed, but attempts to induce the tachycardia were again unsuccessful, even under isoproterenol. However, after delivering a low-watt (20 W), low-temperature (40°) radiofrequency application into the SP region, sustained AVNRT started within 30 s. Successful SP ablation was achieved at higher power/temperature (50 W/55°).

This case underlines the importance of the atrial lead in patients with Brugada syndrome, in view of the higher risk of developing atrial arrhythmias including AVNRT [1]. In our case, the atrial lead did not contribute to arrhythmia discrimination by the device, but had its value in the post-hoc analysis, which prompted the repeated EPS. Misclassification resulted from the high rate (ventricular fibrillation [VF] zone), but would still have occurred in the ventricular tachycardia (VT) zone (‘dual-chamber detection’), as it cannot differentiate AVNRT from VT with 1:1 retrograde conduction (Biotronik, SMART). To avoid further inappropriate shocks, a strategy might be dual-zone programming with a VF zone at even higher rate limits, longer detection counters, and anti-tachycardia pacing (ATP) during capacitator charging (possibility to stop AVNRT before shock delivery), besides a VT zone with ATP as primary therapy and introduction of a morphology criterion (need to program ‘ventricular-only detection’). However, in high-risk patients with Brugada syndrome, as in our case, we are reluctant to increase the VF zone or prolong the VT detection intervals, but would recommend an attempt of ATP during charge and would stress once more that SP ablation should be favoured.

Furthermore, our case points out the issue of AVNRT non-inducibility during EPS, notably in patients with poor ventriculoatrial conduction as in our patient (retrograde AVN Wenckebach CL of 530 ms). The high adrenergic tone during exercise was clearly needed ‘in vivo’ to overcome the retrograde refractory period of the FP after a critical atrium-His interval. Reproduction of sustained re-entry during EPS could only be achieved by delivering of a low hyperthermic radiofrequency current in the AVN area and this obviously by modification in retrograde FP conduction (increase of conduction velocity and decrease of refractory period) [2].
